# Psychosocial and mental health correlates of perceived teacher support among young adults across cultures: above and beyond the effects of childhood trauma

**DOI:** 10.3389/fpsyg.2026.1729702

**Published:** 2026-04-23

**Authors:** Hong Wang Fung, Cherry Tin Yan Cheung, Anson Kai Chun Chau, Guangzhe Frank Yuan, Caimeng Liu, Cindy Sin Yu Leung, Henry Tak Shing Chiu, Edward K. S. Wang, Shan-yan Huang

**Affiliations:** 1 School of Nursing, The Hong Kong Polytechnic University, Kowloon, Hong Kong SAR, China; 2 Mental Health Research Center, The Hong Kong Polytechnic University, Kowloon, Hong Kong SAR, China; 3 Department of Social Work, Hong Kong Baptist University, Kowloon, Hong Kong SAR, China; 4 Department of Psychiatry, The University of Hong Kong, Pokfulam, Hong Kong SAR, China; 5 College of Teacher Education, Institute of Education Science, Leshan Normal University, Leshan, China; 6 Faculty of Medicine and Health Sciences, University of East Anglia, Norwich, United Kingdom; 7 Massachusetts General Hospital, Harvard Medical School, Boston, MA, United States; 8 Department of Marketing and Supply Chain Management, Overseas Chinese University, Taichung, Taiwan

**Keywords:** childhood adversities, post-traumatic stress, post-traumatic stress disorder (PTSD), teacher support, youth mental health

## Abstract

**Background:**

Although individuals typically spend more than 10 years in school, with school days often lasting 6 hours or more, little is known about the potential psychosocial and mental health benefits of teacher support for young people while considering the effects of childhood trauma.

**Methods:**

This study examined whether perceived teacher support would be positively associated with self-esteem and confidence in career development while negatively associated with depressive symptoms and post-traumatic stress (PTS) symptoms, after controlling for childhood trauma. This study analyzed data from two culturally different samples of young adults (*N* = 781 Chinese speakers and *N* = 283 English speakers).

**Results:**

Perceived teacher support was significantly associated with self-esteem (β = 0.282–0.300, *p* < 0.001), confidence in career development (β = 0.324–0.352, *p* < 0.001), and fewer depressive symptoms (β = −0.173 to −0.220, *p* < 0.001) among young adults, even after controlling for age, gender, childhood trauma, and history of seeking mental health services. However, perceived teacher support was not associated with PTS symptoms (*p* = 0.450–0.473).

**Conclusion:**

The findings are very consistent across two samples, despite differences in measures and sociocultural and language backgrounds. The study highlights the potential benefits of teacher support in promoting the well-being of young people, especially in the context of childhood trauma, although further longitudinal studies are needed. The findings point to the need to provide teachers with necessary training, support and resources so that they are ready to support students in need.

## 1 Introduction

The United Nations defines “youth” as individuals between the ages of 15 and 24. Youth is a critical stage of development marked by a variety of challenges and opportunities. According to Erikson (1950), young people need to develop a sense of identity and self-esteem during their early years, and these are important to their well-being and psychosocial development. Youth is also a stage where young people begin to explore their strengths and interests and prepare for their future roles and careers in the society. This stage is developmentally important and it lays the foundation for many aspects of an individual’s future life, career, and overall well-being. In addition, most mental disorders (62.5%–75%) are developed by the age of 25 (Kessler et al., 2007; Solmi et al., 2022). Mental health problems among young people can have profound effects on their social and occupational functioning, which can, in turn, impact the socio-economic development of the society as a whole (Knapp and Wong, 2020). Therefore, it is important to better understand and prevent psychosocial and mental health problems among young people.

The developmental pathway following adversity is essentially heterogeneous. This study is grounded in the Resilience Theory (Mastromatteo et al., 2025). It proposed that some individuals demonstrate positive adaptation and maintain mental health when exposed to adversity. This theory categorizes risk factors that increase the probability of negative outcomes and protective factors that increase the probability of positive outcomes. While there are many biological, psychological and social factors that could be associated with the psychosocial and mental health problems among young people, childhood trauma and adversities (e.g., exposure to violence and maltreatment) have been identified as “the most preventable causes” and risk factors for mental health problems, substance abuse, and health risk-taking behaviors; moreover, adverse childhood experiences are also closely associated with many leading causes of death, such as cardiovascular problems, diabetes, and suicide (The Childhood Adversity Narratives, 2015; Racine et al., 2022; Bertule et al., 2021). Many systematic reviews and meta-analyses have already provided strong evidence for the long-lasting psychological and health impacts of childhood trauma (e.g., Graf et al., 2021; Holman et al., 2016; McKay et al., 2021; Halpern et al., 2018). In other words, childhood trauma is a well-known, major risk factor for psychosocial and mental health problems, including post-traumatic stress (PTS) symptoms. PTS symptoms are one of the important mental health issues among youth, which can result from a range of traumatic experiences. A systematic review showed a strong relationship between childhood trauma (sexual, physical abuse, neglect, traumatic events) and PTSD in adults. Especially, some studies suggested that early-onset and adolescent-onset abuse significantly predicts higher PTS symptoms levels (Umar et al., 2025). Based on developmental psychopathology, childhood trauma in this study refers to early adversity and risk factor for later mental illness. This study hypothesized and examined the association between childhood trauma and the presentation of PTS symptoms in young adulthood, acknowledging that PTS symptoms are distinct clinical construct with complex etiology.

However, much less is known about the potential effects of other social-interpersonal factors on psychosocial and mental well-being while taking into account the effects of childhood trauma. In the current literature, some studies showed that interpersonal stress is related to mental health problems or psychosocial flourishing in the context of childhood adversities (e.g., Stevens et al., 2013; Li et al., 2020). Family resilience or support was also found to be associated with fewer trauma-related mental health problems (Wakhid and Hamid, 2020; Kasler et al., 2008; Gallo et al., 2019). Nevertheless, considering the substantial amount of time that young people spend in school during their formative years, it is surprising that there is a lack of studies examining the potential psychosocial and mental health benefits of perceived social support from teachers as protective factors. Bronfenbrenner’s Ecological Systems Theory provided a crucial framework in conceptualizing the development of young people in a nest of environmental systems (Lubis et al., 2024). We focus on the microsystem that the school, especially their relationship with teachers is the direct environment to provide source of stability, structure, and positive relational experience for children when they had family as a source of risk and adversity. A few studies reported that teacher-student relationships are related to enhanced engagement in school (Quin, 2017). Additionally, teacher burnout, which may influence how teachers interact with students, has been linked to lower quality student motivation and poor academic achievement (Madigan and Kim, 2021). Metheny et al. (2008) also found that perceived teacher support is associated with better career-related self-efficacy and vocational outcome expectations. Yet, no study has explored whether perceived teacher support would be associated with psychosocial and mental health outcomes after controlling for childhood trauma. In modern society, individuals typically spend more than 10 years in school, with school days often lasting 6 hours or more. The experience of interacting with the teachers could profoundly affect the psychosocial development and well-being of the students. Teachers not only could provide support and guidance on academic and career development, but they may also provide psychosocial and emotional support for students in need. It has long been recommended that teachers can play an important role in identifying and supporting students exposed to child abuse (e.g., Hazzard, 1984). There is also an increasing number of studies exploring the potential benefits of teacher-mediated interventions to support traumatized students (Coombe et al., 2015). For example, even when a student was exposed to trauma and adversities, a supportive teacher can comfort the student, offer guidance on coping, make essential referrals for social resources, and can even be a role model for the student to learn how to behave, cope, and manage emotions. Maladaptive coping and emotional avoidance are key predictors of trauma-related responses (Vancappel et al., 2022; Díaz-Tamayo et al., 2022; Yuan et al., 2022). With a supportive teacher, the student might have more opportunities to deal with adversities and the corresponding emotions in a healthy way, without avoidance.

### 1.1 Hypotheses

According to the Resilience Theory and Bronfenbrenner’s Ecological Systems Theory and considering this literature and the research gaps on the potential benefits of perceived teacher support, we hypothesized that perceived teacher support is the protective factor that would be associated with better psychosocial and mental health outcomes among young people, even after controlling for the effects of childhood trauma (the risk factor). In particular, the present study examined whether perceived support from teachers would be positively associated with two specific psychosocial outcomes – namely self-esteem and confidence in career development – while negatively associated with two common types of mental health symptoms – namely depressive symptoms and PTS symptoms – even after controlling for the effects of childhood trauma. PTS symptoms are common responses to childhood trauma and adversities and are of public health concern, and therefore they were included as one of the studied outcomes in this study.

Taking into account the potential impacts of sociocultural factors on trauma and its consequences (Asnaani and Hall-Clark, 2017; de Oliveira Maraldi et al., 2021), teacher-student relationship (Thijs et al., 2012), and psychosocial adjustment (Tsai et al., 2016), and to ensure the generalizability and validity of the findings, we analyzed data from two independent samples of young adults with different language and sociocultural backgrounds. Specifically, we tested our hypotheses in a sample of Chinese-speaking young adults first, and then sought to replicate the findings in a sample of English-speaking young adults.

## 2 Materials and methods

### 2.1 Participants

We analyzed data from two samples, including a sample of Chinese-speaking adults and a sample of English-speaking adults.

Participants in Sample 1 were recruited in a survey project which obtained ethical approval at the Hong Kong Baptist University, Hong Kong. This project recruited young adults from social media platforms in order to examine the relationship between social experiences and well-being in 2023. Participants should be aged between 18 and 24, give informed consent and agree to participate, and can read and write Chinese. Only participants who self-reported with a diagnosis of a reading disorder, dementia or intellectual disabilities were excluded. Since we used an online survey platform to collect the data, no missing data was allowed before submitting the response. The survey took around 15–25 min to complete.

Participants in Sample 2 were recruited in another survey project which obtained ethical approval at the Leshan Normal University, China. This project also recruited young adults from social media platforms to investigate the social correlates of physical and mental health problems in 2023. Participants should be aged between 18 and 24, give informed consent and agree to participate, and can read and write English. Similarly, only participants with a diagnosis of a reading disorder, dementia or intellectual disabilities were excluded. Same as the study in the Sample 1, no missing data was allowed before submitting the response on the online survey platform. The survey took around 15–25 min to complete.

It is worth noting that online technology is now commonly used by researchers to conduct epidemiological and social studies, and web-based measures can also be reliable and valid (Vallejo et al., 2007; Sordo Vieira et al., 2022). In addition, the online surveys used in both samples included attention items to ensure the validity of responses. Participants who failed to answer these questions were excluded from the analysis.

### 2.2 Measures

Participants in both samples completed online surveys that included questions about demographic and health backgrounds. In addition, the surveys also included standardized measures of the following variables in their respective languages.

#### 2.2.1 Perceived teacher support

In Sample 1, it was assessed using a measure adapted from the Multidimensional Scale of Perceived Social Support (MSPSS) (Kazarian and McCabe, 1991; Zimet et al., 1988). In particular, we adapted two items to assess the retrospective perception of perceived support from teachers – i.e., “I can count on my teachers when things go wrong” and “I have teachers with whom I can share my joys and sorrows” (1 = Very Strongly Disagree; 7 = Very Strongly Agree). Given that only 2 items are included, it is not sufficient for a factor analysis. Yet, the face validity was confirmed by a team of external scholars, which included registered social workers, nurses, and Ph.D. students/researchers. This 2-item measure had excellent internal consistency (α = 0.908) in our Sample 1. In Sample 2, we used the 5-item Emotional Support subscale of the Teacher Support Scale (TSS) (McWhirter et al., 2000; Metheny et al., 2008), which is originally a 27-item self-report measure to assess the retrospective perception of perceived support from teachers. Sample items of this subscale include “(most teachers in my high school) enjoy having me in their classes” and “care about what happens to me” (1 = Strongly Disagree; 5 = Strongly Agree). The confirmatory factor analysis revealed a robust single-factor model with satisfactory to strong, significant indicator loadings (0.66–0.91, all *p* < 0.001) that demonstrate excellent validity for the latent construct, supported by an outstanding SRMR of 0.05 and an acceptable CFI of 0.93, indicating a promising foundation despite room for further refinement to improve overall fit indices such as RMSEA and TLI. This 5-item measure was internally consistent (α = 0.839) in our Sample 2.

#### 2.2.2 Childhood trauma

In both Sample 1 and Sample 2, it was assessed using the Brief Betrayal Trauma Survey, which is a commonly-used self-report 24-item measure which assesses 12 different traumatic experiences (e.g., natural disaster, physical abuse, sexual abuse, psychological maltreatment) during childhood and adulthood (Goldberg and Freyd, 2006). The BBTS had good test-retest reliability in both English and Chinese contexts (Goldberg and Freyd, 2006; Fung et al., 2022). Only the 12 items for childhood traumatic experiences were used.

#### 2.2.3 Self-esteem

In both Sample 1 and Sample 2, it was assessed using the single-item measure of self-esteem (SISE). The SISE asked: “How satisfied are you with yourself?” (1 = Very Dissatisfied; 9 = Very Satisfied). The SISE is a valid single-item self-report measure of self-esteem and it is also highly correlated with multiple-item scales of self-esteem (Sawicki–Luiza and Atroszko, 2017; Robins et al., 2001). The Chinese version of the SISE also had good test-retest reliability (Fung et al., 2023).

#### 2.2.4 Depressive symptoms

In both Sample 1 and Sample 2, depressive symptoms were assessed using the Patient Health Questionnaire (PHQ). While the full 9-item PHQ was used in Sample 1, a 3-item version (i.e., Items 1, 2 and 9) of the PHQ was used in Sample 2. The PHQ is a widely-used self-report measure of DSM depressive symptoms with established psychometric properties (Kung et al., 2013; Kroenke et al., 2001), including in the Chinese context (Yeung et al., 2008). A shortened version of the PHQ has also be used in the literature (Manea et al., 2016).

#### 2.2.5 PTS symptoms

In Sample 1, PTS symptoms were measured with the 6-item PTSD subscale of the International Trauma Questionnaire (ITQ), which is originally a 18-item measure of post-traumatic symptoms recognized in ICD-11 and it also had excellent reliability and validity (Cloitre et al., 2018, 2021), including in the Chinese context (Ho et al., 2019). In Sample 2, PTS symptoms were measured with the 4-item version of the PTSD Checklist for DSM-5 (4-item PCL-5), which has been found to be a reliable and valid screening tool (Geier et al., 2020; Blevins et al., 2015).

#### 2.2.6 Confidence in career development

In Sample 1, it was measured using two items adapted from a previous Chinese study (Chui et al., 2017): “I am able to find a career that I love” and “I am able to obtain a job in my chosen career” (1 = Not Confident at all; 5 = Fully Confident). Given that only 2 items are included, it is not sufficient for a factor analysis. However, the face validity of the measure was reviewed and confirmed by a team of external scholars, which included registered social workers, nurses, and Ph.D. students/researchers. This measure had good internal consistency in our study (α = 0.862). In Sample 2, it was measured using the Career Confidence subscale of the Career Resources Questionnaire (CRQ-CC) (Hirschi et al., 2018), which is a validated measure and this subscale includes 4 items (e.g., “I believe that I can successfully manage career-related challenges”) (1 = Not True at All; 5 = Completely True). This measure had excellent internal consistency in our study (α = 0.926).

### 2.3 Data analysis

SPSS 22.0 was used for statistical analyses. We first performed descriptive analyses of the major variables and conducted Pearson and point-biserial correlation analyses to examine the correlates of perceived teacher support. Next, we tested the hypotheses that perceived teacher support would be significantly associated with each of the four psychosocial outcomes even after controlling for childhood trauma and demographic variables. We first tested the hypotheses in Sample 1 and then examined if the findings could be replicated in Sample 2.

## 3 Results

### 3.1 Sample characteristics

Sample 1 included 781 Chinese-speaking participants aged 18–24. Most of them were female (84.0%) and lived in Taiwan (88.3%) and Hong Kong (9.2%); 37.4% of them had completed an undergraduate degree or higher. About half of them (57.9%) used to seek professional help because of emotional or mental health issues. Only 18.6% of them were currently full-time employed.

Sample 2 included 283 English-speaking participants aged 18–24. Most of them were female too (91.2%). This is a regionally diverse sample as these participants lived in 10 different countries/regions, mainly from Canada (25.44%), United Kingdom (22.61%), New Zealand (14.84%), and United States (14.13%). Only a small number of them (17.3%) had completed an undergraduate degree or higher. Most participants (83.0%) had received professional help before because of emotional or mental health issues. Only 15.9% of them were currently full-time employed.

Further details of the sample characteristics are reported in Table 1.

**TABLE 1:** Sample characteristics and correlates of perceived teacher support.

	Sample 1 (*N* = 781 Chinese speakers)			Sample 2 (*N* = 283 English speakers)		
Variables	Possible range	Mean (*SD*)/ percentage	Correlates of perceived teacher support (*r*)	Possible range	Mean (*SD*)/ percentage	Correlates of perceived teacher support (*r*)
Perceived teacher support	1–7	2.52 (1.64)	1	5–25	18.46 (4.44)	1
Age	18–24	21.00 (2.01)	−0.115^**^	18–24	20.04 (1.88)	0.004
Gender (female)	/	84.0%	0.023	/	91.2%	0.043
Ever sought help for mental health problems	/	57.9%	−0.047	/	83.0%	−0.043
Childhood trauma	1–12	2.62 (2.30)	−0.143^***^	1–12	4.35 (2.95)	−0.191^**^
Self-esteem	1–9	3.75 (1.93)	0.325^***^	1–9	4.31 (2.23)	0.332^***^
Depressive symptoms	3–27	14.05 (6.59)	−0.232^***^	0–9	5.34 (2.66)	−0.284^***^
Post-traumatic stress symptoms	0–30	14.09 (8.09)	−0.089^*^	0–16	9.24 (4.39)	−0.144^*^
Confidence in career development	2–10	5.49 (2.39)	0.333^***^	4–20	11.86 (4.38)	0.365^***^

*p < 0.05;

^**^p < 0.01;

^***^p < 0.001.

### 3.2 Relationship of perceived teacher support and psychosocial and mental health outcomes

In Sample 1, correlation analyses revealed that perceived teacher support was positively correlated with self-esteem (*r* = 0.325, *p* < 0.001) and confidence in career development (*r* = 0.333, *p* < 0.001) and negatively correlated with both depressive (*r* = −0.232, *p* < 0.001) and PTSD symptoms (*r* = −0.089, *p* < 0.05).

The same pattern was also observed in Sample 2. Perceived teacher support was positively correlated with self-esteem (*r* = 0.332, *p* < 0.001) and confidence in career development (*r* = 0.365, *p* < 0.001) while negatively correlated with both depressive (*r* = −0.284, *p* < 0.001) and PTSD symptoms (*r* = −0.144, *p* < 0.05).

Further analyses using hierarchical multiple regression revealed that perceived teacher support was also significantly associated with self-esteem (β = 0.282–0.300, *p* < 0.001), confidence in career development (β = 0.324–0.352, *p* < 0.001), and depressive symptoms (β = −0.173 to −0.220, *p* < 0.001) in both Sample 1 and Sample 2, even after controlling for age, gender, childhood trauma and previous mental health help-seeking. Perceived teacher support, however, was not significantly associated with PTS symptoms (*p* = 0.450–0.473) in both samples. It is essential to note that due to the use of different scales, the raw mean scores of the measures are not directly comparable between the two samples, yet the correlation coefficients (Pearson’s *r*) are standardized effect sizes. Therefore, the patterns of relationships between variables can be compared directly across the two groups and the findings are very consistent in both samples (see Table 2).

**TABLE 2:** Hierarchical multiple regression predicts psychosocial outcomes from perceived teacher support after controlling for childhood trauma.

Sample 1 (*N* = 781)	Self-esteem					Depressive symptoms					Post-traumatic stress symptoms					Confidence in career development				
Variables	β	*p*	*F*	Δ*R*^2^	Δ*F*	β	*p*	*F*	Δ*R*^2^	Δ*F*	β	*p*	*F*	Δ*R*^2^	Δ*F*	β	*p*	*F*	Δ*R*^2^	Δ*F*
Step 1			20.035^***^	0.094	20.035^***^			61.391^***^	0.240	61.391^***^			63.112^***^	0.245	63.112^***^			3.347*	0.017	3.347*
Childhood trauma	−0.206	0.000				0.361	0.000				0.366	0.000				−0.095	0.012			
Age	0.036	0.298				0.027	0.395				0.051	0.105				−0.017	0.627			
Gender (Female)	0.030	0.385				0.037	0.243				0.104	0.001				0.006	0.878			
Ever sought help	−0.170	0.000				0.227	0.000				0.207	0.000				−0.059	0.117			
Step 2			34.214^***^	0.087	82.511^***^			57.122^***^	0.029	30.661^***^			50.576^***^	0.001	0.572			20.881^***^	0.102	89.492^***^
Childhood trauma	−0.166	0.000				0.338	0.000				0.363	0.000				−0.052	0.149			
Age	0.066	0.045				0.009	0.764				0.048	0.125				0.015	0.653			
Gender (Female)	0.026	0.430				0.039	0.205				0.104	0.001				0.001	0.974			
Ever sought help	−0.170	0.000				0.227	0.000				0.207	0.000				−0.059	0.098			
Perceived teacher support	0.300	0.000				−0.173	0.000				−0.024	0.450				0.324	0.000			
**Sample 2 (*N* = 283)**	**Self-esteem**					**Depressive symptoms**					**Post-traumatic stress symptoms**					**Confidence in career development**				
**Variables**	**β**	***p***	***F***	**Δ*R*^2^**	**Δ*F***	**β**	***p***	***F***	**Δ*R*^2^**	**Δ*F***	**β**	***p***	***F***	**Δ*R*^2^**	**Δ*F***	**β**	***p***	***F***	**Δ*R*^2^**	**Δ*F***
Step 1			7.711^***^	0.100	7.711^***^			11.641^***^	0.143	11.641^***^			34.811^***^	0.334	34.811^***^			2.178	0.030	2.178
Childhood trauma	−0.291	0.000				0.334	0.000				0.530	0.000				−0.117	0.054			
Age	0.128	0.027				−0.098	0.082				−0.080	0.107				0.108	0.072			
Gender (Female)	0.090	0.120				−0.077	0.169				−0.021	0.671				0.056	0.352			
Ever sought help	−0.062	0.283				0.143	0.012				0.182	0.000				−0.080	0.187			
Step 2			11.815^***^	0.076	25.510^***^			12.973^***^	0.046	15.818^***^			27.004^***^	0.001	0.517			9.686^***^	0.118	38.539^***^
Childhood trauma	−0.235	0.000				0.290	0.000				0.523	0.000				−0.047	0.422			
Age	0.119	0.033				−0.091	0.098				−0.079	0.113				0.096	0.087			
Gender (Female)	0.070	0.206				−0.062	0.259				−0.018	0.710				0.031	0.580			
Ever sought help	−0.057	0.305				0.139	0.012				0.182	0.000				−0.073	0.196			
Perceived teacher support	0.282	0.000				−0.220	0.000				−0.036	0.473				0.352	0.000			

*p < 0.05;

^***^p < 0.001.

An interesting and novel finding to note is that childhood trauma was significantly associated with confidence in career development in Sample 1 (β = −0.095, *p* = 0.012). In Sample 2, this relationship was only marginally significant (β = −0.117, *p* = 0.054) after taking into account the effects of age, gender, and previous mental health help-seeking.

## 4 Discussion

This study is the first to examine the psychosocial and mental health correlations of perceived teacher support after taking into account the effects of childhood trauma in a cross-cultural context. Despite differences in measures and sociocultural and language backgrounds, findings from two independent samples consistently showed that perceived teacher support was significantly associated with self-esteem (β = 0.282–0.300, *p* < 0.001), confidence in career development (β = 0.324–0.352, *p* < 0.001), and depressive symptoms (β = −0.173 to −0.220, *p* < 0.001) – but not with PTSD symptoms (*p* = 0.450–0.473) – among young adults, even after controlling for age, gender, childhood trauma, and history of seeking mental health services. The findings and their implications require further discussion.

First of all, our findings point to the potential benefits of providing teacher support for the psychosocial well-being and development of young people. While childhood trauma was strongly associated with lower self-esteem and more depressive symptoms, perceived teacher support was the strongest predictor for self-esteem in both samples. More importantly, perceived teacher support was also significantly and negatively associated with depressive symptoms, even after controlling for childhood trauma. This highlights the potentially important role that teachers can play in supporting young people, even when they have experienced trauma and present with mental health problems. Our findings support recent literature on teacher-delivered interventions for students with mental health needs (Shelemy et al., 2020), although further evaluation studies are required.

Additionally, our findings align with recent advocacy for trauma-informed schools (e.g., Wiest-Stevenson and Lee, 2016; Mendelson et al., 2015). If teachers are more familiar with trauma psychology and mental health, they could be more capable of identifying and supporting students who need help. Therefore, more training for teachers may be beneficial, and future studies should also evaluate whether trauma-informed care training for teachers could improve students’ outcomes.

Furthermore, burnout has long been a common problem among teachers (García-Carmona et al., 2019). Many teachers are extremely busy and fully occupied with paperwork (Tsang, 2018), leaving them very limited time to interact with students. In the sake of the well-being of students, and considering our findings that teacher support may be helpful for students to deal with psychosocial and mental health challenges, it is important to address problems within school systems and ensure the well-being of teachers. Teachers should be provided with more knowledge, time and resources so that they could be ready to support students. Social workers, psychologists and other mental health professionals should work more closely with teachers to promote the well-being of young people too. Since early identification and timely intervention are important, universal screening for psychosocial and mental health problems is recommended (Albers et al., 2007). In this regard, teachers should be more ready to recognize and support those students who present with psychosocial and mental health problems, and should have more support from mental health professionals.

Another point worth noting is that perceived teacher support was not significantly associated with fewer PTS symptoms. This may be because PTS and depressive symptoms are distinct problems with potentially different predictors (Lommen et al., 2025). Besides, it can be explained by the multifactorial etiology of PTS symptoms. Our sample may experience more recent traumatic events, for example, assaults, car accidents, bullying at workplace, which could not be possibly buffered by teacher support. This emphasizes the complexity of trauma and the need of support throughout lifespan. It may also mean that teacher support is not sufficient to buffer the effects of trauma, as other types of social environments (e.g., family support, toxic stress) may be more influential to PTSD symptoms (Bisson, 2009; Charuvastra and Cloitre, 2008; Wang et al., 2021). Given that young people often spend a considerable amount of time in school, further research is needed to explore which school-related factors may help traumatized young people manage their psychophysiological reactions to trauma and extreme stress.

To our knowledge, this is also the first study to examine the potential effects of childhood trauma on confidence in career development. Our findings indicate that the association between childhood trauma and confidence in career development was statistically significant in Sample 1 but only marginally significant in Sample 2. As childhood trauma may impact both mental health and educational outcomes (Romano et al., 2015), which could subsequently affect career development, further studies are required to explore the extent to which childhood trauma is associated with poor career-related outcomes.

Although there are some interesting findings with potential implications for researchers, practitioners and policymakers concerning the well-being of young people, this study had several limitations. First, the cross-sectional nature of the data did not allow us to reveal the causal relationship between the variables, thus future longitudinal studies are required. Second, although we used reliable and valid standardized measures, this study relied on self-report data. Without conducting structured interviews, the mental health status of the participants could not be confirmed. In addition, the use of short and/or single-item measures are sometimes regarded as a limitation. Despite their bad reputation, short and single-items are in fact well validated and widely used in epidemiological studies in the psychology and mental health fields, and its use is supported by the literature (for an overview, see Allen et al., 2022). Short or single-item measures of self-esteem (Robins et al., 2001), career-related outcomes (Huang et al., 2026), and mental health (Stubbs and Achat, 2023), for example, as used in the present study, are also widely used and supported in the literature. Nevertheless, we should acknowledge that, given that this is a simply cross-sectional survey study, we did not pilot test the measures prior to the actual study. Future studies shall replicate our results using more comprehensive assessment tools. Third, we analyzed data from convenience samples recruited online, which may be affected by self-selection bias; besides, most participants were female, which may be because of the use of online recruitment (Whitaker et al., 2017). Therefore, the generalizability of our findings may be limited. However, we would like to note that opportunistic convenience samples are widely used to explore relationships among variables in mental health and epidemiological studies, and representative samples are not typically necessary unless we want to estimate prevalence rates (Tyrer and Heyman, 2016). Forth, the help-seeking rate of our participants (57.9% in sample 1 and 83% in sample 2) is much higher than the epidemiological data (e.g., ≤5%–10% young adults with mental health symptoms use mental health services in the past 6–12 months) (Mori et al., 2024), indicating that the online recruitment methods likely resulted in self-selecting bias. Fifth, since the assessments of perceived support from teachers were retrospective, this may also involve potential recall bias. Nevertheless, this study had an important strength: We replicated the findings in two independent samples with different sociocultural and language backgrounds – the results are very consistent across samples. The use of different measures in the two samples can be seen as a limitation. As a result, we cannot draw any direct cross-cultural comparisons. Yet, it also shows that our results are consistent across two samples despite the use of different measures. It demonstrates that our findings could be replicated.

## 5 Concluding remarks

This study found that perceived teacher support was associated with better self-esteem, greater confidence in career development, and fewer depressive symptoms, even after controlling for the effects of childhood trauma. The findings are consistent across two samples with different cultural and language backgrounds, highlighting the potential benefits of teacher support in promoting the psychosocial well-being of young people. Teachers may play an important role in supporting young people, even when they have experienced childhood trauma and present with mental health problems, and it is important to provide teachers with the necessary support and resources to effectively support students in need.

## Data Availability

The raw data supporting the conclusions of this article will be made available by the authors, without undue reservation.
